# *Ex vivo *effects of flavonoïds extracted from *Artemisia herba alba *on cytokines and nitric oxide production in Algerian patients with Adamantiades-Behçet's disease

**DOI:** 10.1186/1476-9255-8-35

**Published:** 2011-11-21

**Authors:** Djamel Messaoudene, Houda Belguendouz, Mohamed Laid Ahmedi, Tarek Benabdekader, Fifi Otmani, Malika Terahi, Pierre Youinou, Chafia Touil-boukoffa

**Affiliations:** 1 Laboratoire de Biologie Cellulaire et Moléculaire (LBCM), FSB, USTHB. Université de Bab-Ezzouar. BP32, 16111. Algiers, Algeria; 2 Département de Biologie, Faculté des sciences, université de Boumerdes, Algeria; 3 Service de médecine Interne, CHU Mustapha Bacha. Algiers, Algeria; 4 Service d'ophtalmologie, CHU Bab El Oued. Algiers. Algeria; 5 Laboratoire d'immunologie. Centre Hospitalier Universitaire. Brest, France

**Keywords:** Adamantiades-Behçet's disease, *Artemisia herba alba*, Flavonoïds, Immunomodulation, IL-4, IL-12, nitric oxide

## Abstract

**Background:**

Adamantiades-Behçet's disease (ABD) is a chronic multisystemic inflammation with unknown pathophysiology. This disorder is associated with a dysregulation of the cytokine network that hyperactivates neutrophils and macrophages. In this study, we investigate the modulatory effects of flavonoïd compounds extracted from Algerian medicinal plant *Artemisia herba alba *on Th1 and Th2 cytokines and nitric oxide production.

**Methods:**

The modulatory effects of flavonoïds extracted from *Artemisia herba alba *on cytokines and nitric oxide production by peripheral blood mononuclear cells isolated from Algerian ABD patients and healthy controls were respectively measured by means of ELISA assays and Griess modified method.

**Results:**

Our results show that flavonoïds significantly reduce the production of interleukin-12, the key effector of T helper 1 (Th1) cells and nitric oxide in a dose-dependent manner in Adamantiades-Behçet's disease. In contrast, the production of IL-4, the key marker of Th2 cells was increased.

**Conclusion:**

This study suggests that *in vitro *supplementation with flavonoïds extracted from *Artemisia herba alba *could have potential immuno-modulatory effects characterised by a down-regulation and up-regulation of Th1 and Th2 cytokines, respectively. Moreover, flavonoïds may prevent nitric oxide induced damages.

## Background

Adamantiades-Behest's disease (ABD) is an inflammatory multisystemic disorder involving mucocutaneous, ocular, arthritic, vascular and central nervous systems. It is most prevalent in the Mediterranean countries, including Algeria, and along the Silk Route. Various factors have been reported contribute to the development of the lesions associated to the disease such as, the genetic susceptibility, environmental factors, anomalies in the inflammatory responses and immune system dysfunction [1,2].

In response to antigens, mediators such as cytokines and chemokines are produced by various cell types, either hematopoietic or non hematopoietic, These mediators orchestrate the immune response by recruitment and activation of different cell types. The involvement of cytokines and chemokines in ABD pathogenesis is reflected by the increase of their concentrations in sera of patients with ABD and some of these mediators correlate with the clinical activity of the disease. Many studies have indeed reported high sera levels of tumor-necrosis factor (TNF)-α, TNF receptor, soluble IL-2R and multiple interleukins (IL-1, IL-6, IL-8, IL-12) [3]. Among them, IL-12 is known to play a major role in the polarization of T helper (Th)1-type cells and sera IL-12 and interferon (IFN)-γ levels are elevated in ABD [4,5]. Moreover, the increase of IL-12 levels in the peripheral blood mononuclear cells (PBMCs) of patients with ABD have been described [6]. This cytokine is responsible for the development of a Th-1 type response and may play a crucial role in the pathogenesis of the disease [7]. However, other investigators have reported increased sera levels of Th2-type cytokines, including IL-4, IL-10, and IL-13 in ABD patients [8], suggesting disturbed cytokines production in ABD. Such dysregulation in cytokine release contributes to the regulation of several enzymes such as the inducible nitric oxide (NO) synthase (iNOS). The function of NO has been delineated in a variety of inflammatory processes. An excess of NO production or peroxynitrite radical could indeed cause oxidative damages through its action on membrane lipids, DNA, proteins and lipoproteins [9,10]. These reactions have functional consequences which may be deleterious [11,12]. The large amounts of NO production have been shown to be correlated with pathophysiology in a plethora of diseases and inflammation processes, such as bowel inflammatory disease [13] and Adamantiades-Behçet's disease [14]. Consequently, the development of molecules aimed to prevent the overproduction of NO constitutes an interesting area of research of a new treatment of chronic inflammatory diseases [15-18].

In the absence of curative treatments in ABD, some patients adopt alternative medicine to avoid the irreversible effects of corticotherapy. For example, *Artemisia herba-alba *(Asteraceae) known as "desert wormwood", or "Chih" as it is commonly named in Algeria is largely consumed. *Artemisia herba-alba *is a plant of the Lamiacaea family, growing in arid and semi-arid climates and it is widely used in folk medicine in different countries. It is characteristic of the steppes and deserts of the Middle East, North Africa, Spain and North western Himalayas [19]. Artemisia has been a productive genus in the search for new biologically active compounds. Phytochemical investigations have proven that this genus is rich in terpenoids, flavonoïds, coumarins, acetylenes, caffeoylquinic acids and sterols and it was shown that Artemisia has multiple beneficial bioactivities: anti-malarial, anti-viral, anti-tumor, anti-pyretic, anti-hemorrhagic, anti-coagulant, anti-anginal, anti-oxidant, anti-hepatitis, anti-ulcerogenic, antispasmodic and anti-complementary activities [20-26].

The flavonoïds detected in *Artemisia herba alba *show also a structural diversity starting from common flavonoïds (flavones glycosides and favonols) to the methyled flavonoïds which is very unusual [27,28]. Some beneficial bioactivities of flavonoïds have been proved, such as antibacterial, anticarcinogenic, antioxidant, antimutagenic, anti-inflammatory, activities and immunomodulatory activities [29-34]

In the present work was investigated the effect of the flavonoïds extracted from the medicinal plant A. *herba alba *on the production of IL-12 and IL-4 and we examined nitric oxide production as a marker of the inflammatory response in the PBMC of patients with Adamantiades-Behçet's disease (ABD). *Artemisia herba alba *may represent an alternative therapy for Algerian patients with ABD.

## Methods

### Patients and controls

Samples from Twenty patients (8 men and 12 women) were obtained from the ophthalmology and internal medicine service, Bab El Oued Hospital and Algiers Medicinal University Hospital (Mustapha Bacha), respectively. Patients with ABD (females and males) were tested during the clinically active stage. The mean age of the active stage was 38.43 years (20-58 years) and the mean duration of the disease was 7.69 years (1-18 years). ABD was diagnosed according to the criteria defined by the international study group for ABD set up in 1990 [35]. All ABD patients were showing the major symptoms including uveitis, aphtosis, articular and neurological manifestations and they had been treated with colchicine and other oral medication (methylprednisolon, cyclophosphamid). Clinical characteristics of ABD patients were given in Table 1. Each patient has given a written informal consent for the study required by the ethic committee of the national agency of research development in health (ANDRS) which supported our project. The healthy controls consisted of 8 males and 12 females (mean age 39.7 years, range 20-59).

**Table 1:** Characteristics of active stage of Adamantiades-Behçet's disease patients (number mean ± standard deviation, percentage)

Sex (M/F)	8/12
Age at disease onset (mean years +SD)	34 ± 10
Follow-up duration (years)	7.69 ± 8.5 (1-18)
Uveitis	7/20 (35%)
Aphtosis	6/20 (30%)
Articular symptoms	4/20 (20%)
NeuroBehçet	3/20(15%)
Treatments	Colchicine, methylprednisolon, cyclophosphamid

### Plant materials and flavonoïds extraction

The flowering aerial parts of A. *herba alba *were collected from Djalfa region (city of south Algeria). The plant was then identified in the department of botany of the national institute of agronomy in Algeria. Flavonoïds were extracted according to the extraction method described previously by Paris and Nothis [36]. Briefly, 20 g of the pulverized plant material were macerated for 24 hours in methano-containing water (7:3). The filtrate was evaporated at 40°C to get completely rid of the solvent mixture. The solid extract was then submitted three times to 50 ml n-butanol to collect the flavonoïds mixture. The solution was filtrated and evaporated at 40°C and then dissolved in water. The extracts were kept frozen (-20°C) until used.

### PBMC cultures

PBMCs were separated by centrifugation on Ficoll-hypaque gradient and washed twice in phosphate-buffered saline, pH 7.2. Cells were then harvested for test viability with trypan blue then resuspended in complete medium consisting of RPMI-1640 supplemented with 10% fetal- calf serum, 100 units/ml penicillin and 100 μg/ml streptomycin.

To test cytokines and NO production, PBMC of ABD patients were treated with different concentrations of flavonoïds (5, 10, 20, 30, 40 or 50 μg/mL) and incubated at 37°C and 5% CO_2 _during 20 hours. Cells were then harvested for test viability and cultures supernatants were conserved at -70°C for cytokines and NO measurements.

For healthy controls and ABD control (before flavonoïds treatment), PBMCs were pre-activated with phytohaemagglutinin (PHA) (5 μg/mL) in 5% CO_2 _at 37°C during 20 hours to mimic the pre-activated stage of ABD cells.

### Cytokine analysis

The concentrations of IL-12 and IL-4 were measured using enzyme linked immunosorbent assays (ELISA) according to manufacture's instructions (Amersham Pharmacia, England). Supernatants samples were added to appropriate wells of a microtiter-plate coated with a specific monoclonal antibody (mAb) against distinct epitopes of IL-12 or IL-4. After incubation for 2 hours, 50 μL of anti IL-12 mAb or anti IL-4 mAb conjugated to horseradish-peroxidase were added. The coloration reaction was read at 540 nm. A standard curve was used to quantify supernatants levels of IL-12 and IL-4. The lowest level of sensitivity was 10 pg/mL for IL-12 and 5 pg/mL for IL-4 of the cytokine.

### NO production by PBMCs

PBMCs of patients and NCs were cultured at 5 × 10^6 ^cells/uL (100 uL/well) with 100 uL of flavonoïds extract (5, 10, 20, 30, 40 or 50 μg/mL) in 96-well microtiter-plates in a humidified incubator at 37°C and 5% CO_2 _for 20 hours. Then NO production was assessed by the determination of the final products of NO oxidation. After reduction of nitrates (NO^-^_3_) by nitrate reductase containing *Pseudomonas oleoveorans *Bacteria (ATCC, 8062) containing nitrate reductase, total nitrite (nitrite NO^-^_2_+ nitrate NO^-^_3_) was determined with the spectrophotometrically Griess reaction as described by Amri *et al *[37]. Griess reagent 2% p-amminobenzene sulphanamide in 5% phosphoric acid and 0.2% N (1-naphhtiyl) ethylene diamine (dihydrochlorid) was added to the sample. The mixture was incubated for 10 minutes at room temperature and the absorbance at 543 nm was read by spectrophotometer. The concentration was determined with reference to a sodium nitrites NaNO_2 _standard (0-200 μmol/mL) curve. Results were expressed as μM of nitrites in supernatants of PBMC cultures.

### Statistical analysis

Results were expressed as the mean ± standard deviation. Statistical differences were assessed using one-way ANOVA with posthoc test of the means according to Tukey's method. In single mean comparisons, Student's t-test was used to test the data and considered statistically significant for *P *values < 0.05. Results and graphics were performed with STATISTCA v. 5 software under windows.

## Results

### *In vitro *production of cytokine during the active stage of ABD

**Figure 1:**
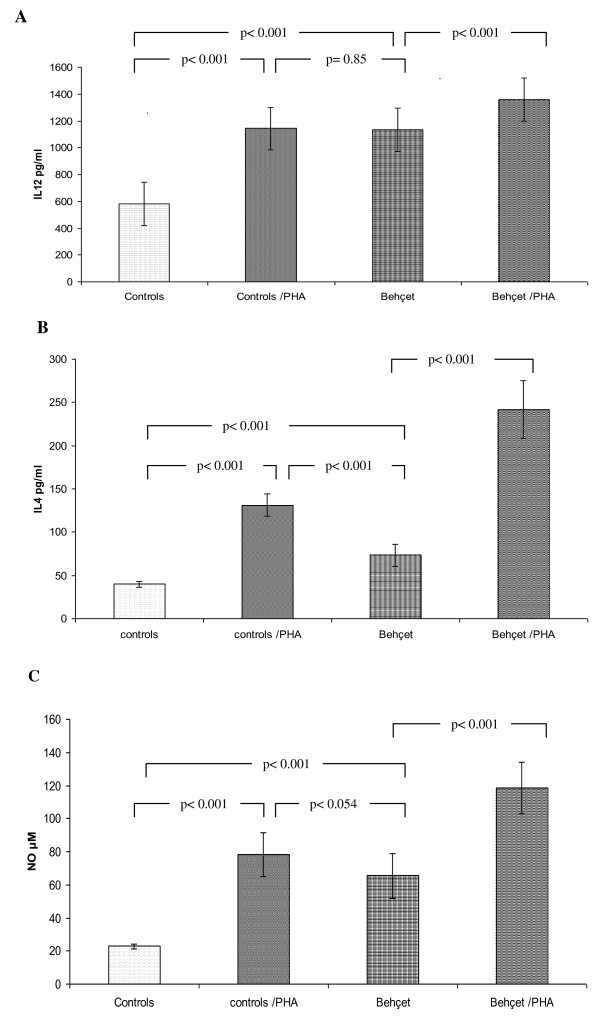
**Cytokines and nitric oxide concentration in PBMC supernatants cultures**. PBMC (5 × 10^6 ^cells/ml) of patients with ABD and healthy controls were cultured with or without 5 μg/ml phytohemagglutnin (PHA) for 20 h. Supernatants were collected and the production level of lL-12 **(A) **and IL-4 **(B) **was determined by a sandwich ELISA. Values shown are mean ± S.D.*p < 0.001 was significantly different from the control value. **C**. Concentration of nitric oxide in the supernatants of culture of PBMC from patients with Adamantiades-Behçet's disease and healthy controls. Cells were treated with 5 μg/mL of PHA. Supernatants were collected after 20 h and the nitrite level was determined by modified Griess reaction. The data represent the mean ± S.D. of cultures. *p < 0.05. NO levels were significantly different from the control values.

To quantify the spontaneous production of IL-12, IL-4 and NO during the active stage, we measured their levels in cultures supernatants of PBMC of ABD patients compared with NCs. As shown in Figure 1A, IL-12 levels in ABD patients were higher than in NCs: 1134.02 ± 83.70 versus 583.02 ± 98.44 pg/mL, p < 0.05. The stimulation with flavonoïds showed an increased level of IL-12 in both ABD patients and NCs (1358.63 ± 118.41 versus 1143.27 ± 104.73 pg/mL, respectively). However, we did not observe any significant difference (P > 0.05). In the absence of PHA stimulation, PBMC from ABD patients showed similar level of IL-12 (1134.03 ± 83.69) compared to PBMC from controls after stimulation with PHA (p < 0.85). This result prompted us to use for the same plant extract treatment experiment the preactivated PBMC from controls and those from ABD patients without activation with PHA.

Quantitative determination of IL-4 in supernatants of ABD patients and normal control's indicated different profiles according to the disease evolution (Figure 1B). Indeed, during the active phase, we observed a higher spontaneous production in ABD patients' PBMC culture supernatants in comparison to the healthy controls (63.1 ± 37 versus 39.7 ± 13.1 pg/mL, P < 0.05). PHA induced a significant increase in the cytokine production in all groups tested. However, IL-4 levels in PBMCs supernatants, after stimulation with PHA (5 μg/mL) were significantly higher in ABD patients compared to the controls (241.8 ± 33.5 versus 131.3 ± 12.6 pg/mL, *p *< 0.001) (Figure 1B). In contrast, the preactivated PBMC from controls showed a significant modification in IL-4 production after treatment with PHA at 5 μg/mL compared to ABD patients without stimulation (*p *< 0.001).

### *In vitro *production of NO during the active stage of ABD

NO measurement in culture supernatants showed that the spontaneous production was higher in ABD PBMC cultures compared to NCs (65.39 ± 15.56 versus 22.84 ± 1.40 μM, *p *< 0.001). Further, NO levels increased significantly in all culture supernatants after treatment with PHA (P < 0.05). We noticed that NO levels in treated PBMC cultures from ABD was higher than in healthy controls (118.48 ± 15.49 versus 78.31 ± 13.41 μM, *p *< 0.001) (Figure 1C). The preactivated PBMC cultures from NCs treated with PHA did not show any significant difference compared to those from ABD patients without prestimulation (*p *= 0.054).

### Flavonoïds did not affect cells viability

To assess if there is any cytotoxic effect of flavonoïds, we tested cell viability before and after PHA treatment. Viability of cells was about 90% before and about 70% after experiments with no differences between flavonoïds-treated and untreated control cells. So flavonoïds were not cytotoxic which is consistent with the previous observations [38].

### Flavonoïds modulate IL-12 and IL-4 production in PBMCs of ABD patients and NCs

**Figure 2:**
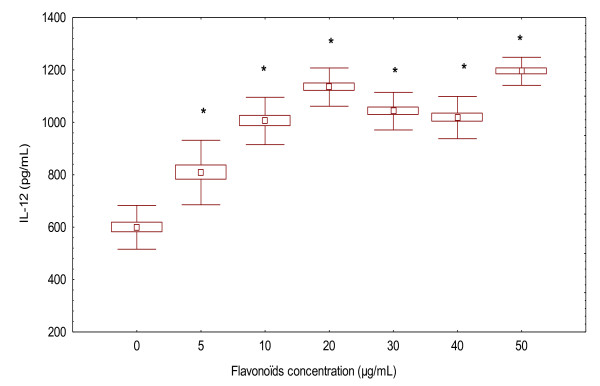
**Effect of flavonoïds on IL-12 production by PHA pre-activated peripheral blood mononuclear cells**. After washing with medium, various concentrations of flavonoïds (5 -50 μg/mL) was added for a period of 20 h. Supernatants were collected and the levels of IL-12 were determined by ELISA. The data represent the mean ± S.D. of triplicate cultures. *p < 0.001, IL-12 levels are significantly different from the control value.

To further confirm the enhancement of the production of the cytokines production by flavonoïds and their aptitude to respond to the PHA preactivated PBMC in healthy controls, flavonoïds were added at different doses 5, 10, 20, 30, 40 or 50 μg/mL for 20 hours. The contents of the wells were centrifuged and kept frozen until analyzed. We observed that flavonoïds did not reduce the IL-12 production in the PBMC stimulation by PHA in NCs (Figure 2). No reversal effects were noticed at any flavonoïd concentrations used. (808.57 ± 123.12 pg/mL, 5 μg/mL of flavonoïds) and (1194.87 ± 53.56 pg/mL, 50 μg/mL of flavonoïds) compared to control values in the absence of flavonoïds (599.47 ± 83.56 pg/mL).

**Figure 3:**
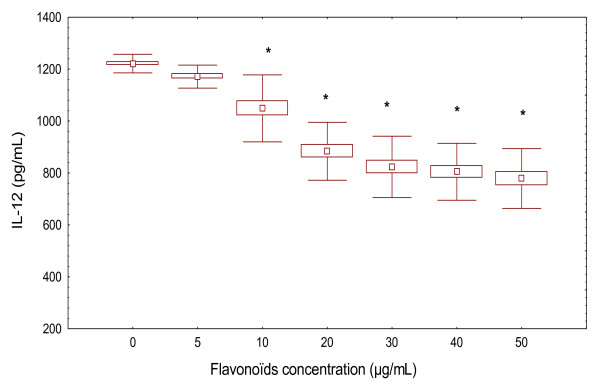
**Effect of various concentrations of flavonoïds extracted from A. *herba alba *on IL-12 production in PBMC (5 × 10^6 ^cells/mL) of patients with ABD**. Presence of cytokines in supernatants was assessed by ELISA. Results are mean ± SD of separate experiments performed in triplicate. *p < 0.05 were significantly different from the control values.

To test if flavonoïds could induce cytokines modulation in patients without PHA, PBMC from patients were cultured in the presence of different concentrations of flavonoïds (5-50 μg/mL). We observed a significant decrease in IL-12 production in a dose-dependent manner (*p *< 0.001). Interestingly, we have observed that the pre-treatment by flavonoïds inhibited IL-12 production (1048.89 ± 128.93 pg/mL with 10 μg/mL of flavonoïds) and (778.63 ± 115.21 pg/mL with 50 μg/mL of flavonoïds) compared to control values (1221.42 ± 36.01 pg/mL). (Figure 3). There is no statistical differences between the doses of flavonoïds (30, 40, 50 μg/ml) on IL-12 production in PBMC from ABD patients.

**Figure 4:**
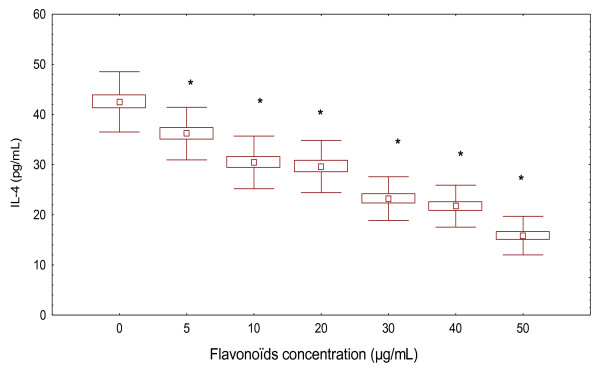
**Effect of flavonoïds on IL-4 production in PHA-stimulated PBMC of healthy controls**. Amounts of IL-4 were measured by ELISA. PBMC (5 × 10^6 ^cells/mL) were cultured for 20 h in the absence or presence of flavonoïds after stimulation with PHA (5 μg/mL). Data represent the mean ± SD of three independent experiments in each sample compared to controls value and PHA-treated alone value (ANOVA with post-hoc test).

Similarly, the amounts of IL-4 released into supernatants of PBMC from controls subjects after pre-stimulation with PHA were determined by ELISA (Figure 4). Treatment of PBMC by different concentrations of flavonoïds inhibited IL-4 production (73.26 ± 10 pg/mL, 30 μg/mL of flavonoïds) and (89.90 ± 13.25 pg/mL, 50 μg/mL of flavonoïds) compared to the control values in the absence of flavonoïds (55.87 ± 7.98 pg/mL).

**Figure 5:**
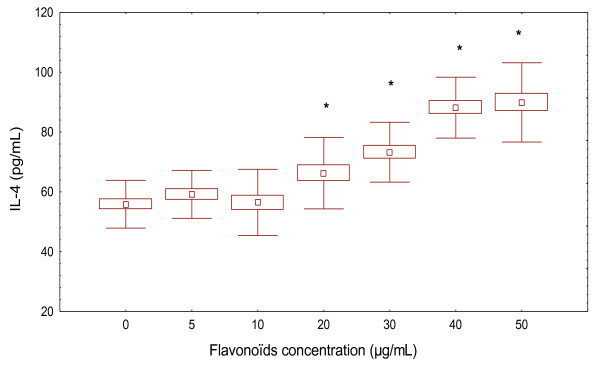
**Effect of flavonoïds extract on IL-4 (pg/mL) production in PBMC of ABD patients (*n *= 20)**. Cells (5 × 10^6 ^cells/mL) were treated with different concentrations (5, 10,20,30,40 and 50 μg/mL) of flavonoïds during 20 h. Presence of cytokines in supernatants were measured by ELISA test. Results are mean ± SD of seven separate experiments performed in triplicate. *p < 0.05 IL-4 levels were significantly different from the control value.

In PBMC from ABD patients, flavonoïds stimulated IL-4 production in a dose-dependent manner and at significantly greater levels compared to the controls (Figure 5). The highest concentration tested (50 μg/mL) exhibited an increased bioactivity. Treatment of flavonoïds induced IL-4 production (1.116 ± 0.207 pg/mL with 10 μg/mL of flavonoïds) and (0.24 ± 0.060 pg/mL with 40 μg/mL of flavonoïds) compared to the control values in the absence of flavonoïds (55. 87 ± 7.98) (Figure 5).

### Flavonoïds inhibited nitric oxide production in PBMC from ABD patients

**Figure 6:**
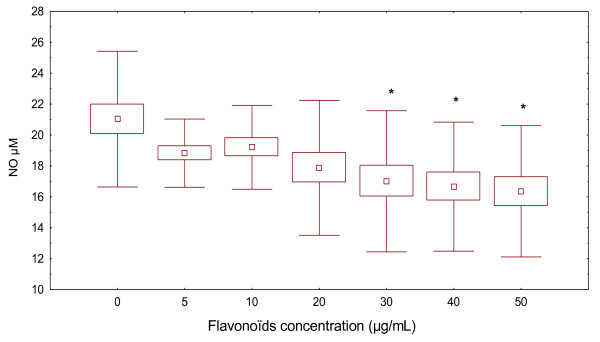
**Effect of flavonoïds on nitric oxide production in PHA-stimulated PBMC of healthy controls**. PBMC (5 × 10^6 ^cells/mL) were stimulated with PHA then cultured with or without flavonoïds (5, 10, 30, 40 and 50 μg/mL). The cell-free supernatants were collected and NO concentration was determined by Griess modified method. The data represents the mean ± S.D. of triplicate cultures.* *p *< 0.05 NO rates was significantly different from the control value (ANOVA with post-hoc test).

Next, we examined the effect of flavonoïds on NO production in PBMC from controls subjects stimulated by PHA were tested. NO levels were measured by Griess modified method. We observed that the treatment did not modulate NO production. As shown in Figure 6, flavonoïds had no statistically significant effect (19.21 ± 2.61 μM with 10 μg/mL of flavonoïds and 16.36 ± 4.25 μM with 50 μg/mL of flavonoïds). The control values in the absence of flavonoïds being 21.03 ± 4.31 μM.

**Figure 7:**
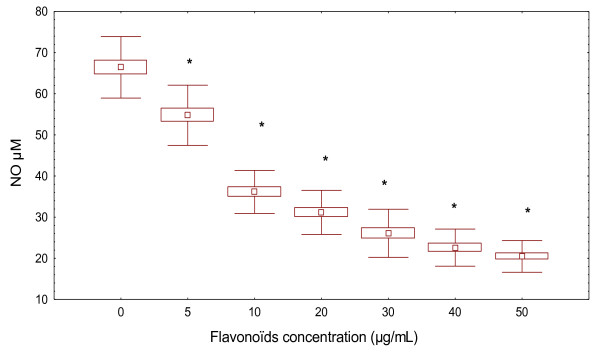
**Effect of different concentration of flavonoïds on nitric oxide production by PBMC in patients with ABD**. Flavonoïds extracts from A*. herba alba *are used at the indicated concentrations and compared to the controls. Supernatants were collected to determine the amount of NO. The data represents the mean ± S.D. of triplicate cultures. *p < 0.05 was significantly different from the control value (absence of flavonoïds).

We then tested the inhibitory effect of flavonoïds on NO production in PBMC from ABD patients (Figure 7). Interestingly, we observed that the treatment with flavonoïds during 20 h reduced the NO concentration in all cultures supernatants (p < 0.05). This inhibitory effect was in dose-dependent manner (10 μg/mL and 50 μg/mL). The corresponding nitrite concentrations assessed were respectively: 36.13 ± 5.22 μM and 20.47 ± 3.85 μM

## Discussion

It is currently recognized that Th cells may be divided into several functional subclasses, Th-1, Th-2, Treg, Th17 cells, based on the production profile of cytokines and their effects on cell mediated and humoral immunity. Th-1 cells produce IL-12, IFN-γ and enhance cell-mediated immunity. Th-1 cells also can inhibit cell-mediated immunologic activities. In our studies, we showed a significant increase of IL12 levels in supernatant of PBMC culture from ABD patients. IL-12 is an immunoregulatory cytokine regulating cell-mediated immune response by inducing the differentiation of uncommitted CD4 Th cells towards type 1 phenotype and a potent cofactor for stimulating the proliferation of differentiated Th1 cells and IFN-γ synthesis [39]. In our study, we confirmed that IL-12 production by PBMC is significantly higher in ABD patients compared to healthy controls suggesting that IL-12 is involved in the pathogenesis of ABD.

Moreover, Th-2 cells produce IL-4, IL-5 and IL-13 and upregulate humoral immunity [40]. In the current study, higher concentrations of IL-4 were also observed in ABD patients. This Th-2 derived cytokine is primarily involved in the activation of B cells, the promotion of growth and the survival of T cells, the inhibition of macrophage and the activation and suppression of Th-1 cells. Recent studies have showed that IL-4 and IL-12 play a significant role in the regulation of the immune responses by their reciprocal antagonistic mechanisms.

We found that the concentration of nitric oxide in the PBMC supernatant were significantly elevated in ABD patients compared to the healthy controls. Here, we postulated that NO could play an important role in the inflammatory process associated with Adamantiades-Behçet's disease [41]. Several studies have suggested that the overexpression of either inducible NO and proinflammatory cytokines might be intimately involved in the pathogenesis and the evolution of ABD [12,42]. An increase in the concentration of NO during the ABD was reported in several studies and this in both the sera of patients [43] and also in the synovial liquid [44]. The presence of NO was also observed in uveitis associated with ABD in particular in the aqueous humour [45,46]. The increase of NO levels in all cases was correlated with the active stage of the ABD.

Stimulation of PBMC cultures from ABD patients with PHA induced an increase of IL-12, IL-4 and NO production. We suggest that the increase of the IL-4 levels in ABD patients after PHA stimulation is probably related to the presence of some factors induced by PHA in PBMC cultures acting on Th-2 cells subset. This purpose remains to be clarified in adequate experiment model. Regarding to the comparison between the production of IL-4 by PHA in healthy controls and ABD patients, the difference observed is probably in relation with the difference in the initial activation level of PBMC state in the two groups of subjects.

Moreover, the increase IL-12 levels after stimulation with PHA on PBMC from ABD patients is related to the production of IFN-γ by Th1 cells. This is consistent with the fact that IFN-γ is known to strongly activate the monocyte/Macrophage system which is the major source of IL-12. Several studies have reported that NO is upregulated by IFN-γ. Recently, our group showed the pivotal role of IFN-γ in pathophysiology of ABD particularly via the NO pathway [46].

There is an increasing interest in herbal medications especially for diseases like ABD [47,48]. The present study demonstrates that flavonoïds extracts from A. *herba alba *highly inhibited the production of the proinflammatory cytokine IL-12 in ABD patients PBMC. The mechanism involved remains to be clarified. Furthermore, in our study we reported that the inhibitory effect on IL-12 production was not due to the toxicity of flavonoïds on PBMC. In fact, in our culture system the use of a high flavonoïds concentration at 50 pg/ml after 20 h incubation yielded almost 70% viable cells. It has been shown that increased IL-12 levels and Th1 cytokines did occur in patients with ABD and have been associated with the pathogenesis.

In contrast to IL-12, we found that flavonoïds promoted a significant increase in IL-4 produced. IL-4 is one of the Th-2 cytokines which has been associated with an improvement in the inflammatory diseases [49]. In the study reported by Koteswara Rao et *al*., [50], flavonoïds have been shown to inhibit extensively the proinflammatory cytokines like TNF- α, IL-12 in a dose-dependent manner. These authors suggested that flavonoïds mediate differentiation from Th-1 to Th-2 cell types and our results are consistent with this study. We also suggest the role of other cytokines or immunoregulatory mediators in the differential regulation of IL-4 (upregulated) and IL-12 (downregulated). These suggestions remain to be clarified in an adequate experimental model. However, it is possible that the inhibition of IL-12 production may be partially mediated by the action of flavonoids through IL-4 induction as both IL-4 and IL-12 have shown to have antagonism effects. IL-4 exerts strong inhibition on Th1-mediated inflammatory processes involving the regulation of the synthesis of inflammatory cytokines (IL-2 TNF-α, IL-1β) and chemokines (CXCL8, CXCL10, CCL2). The effect of flavonoïds on cytokine modulation constitutes a very exciting finding for their possible therapeutic applications.

For the role of NO, we suggest that flavonoïds regulate not only the balance Th1/Th2 towards Th-2 but also NO production. The results presented here show that flavonoïds isolated from A. *herba halba*, affect also NO production in PBMC isolated from patients with ABD in a dose-dependent manner. The inhibitory activity could be resulted from the inhibition of iNOS expression and/or its activity.

## Conclusion

We report here the evidence that the Th-1 cytokines (IL-12) and NO are involved in the pathogenesis of ABD. Our limited follow-up study also suggests that flavonoïds extracts from A. *herba alba *have an effect on the inhibition and the stimulation of the production of IL-12 and of IL-4, respectively. This constitutes a way to switch the immune response from Th-1 to Th2. Further investigations will focus on the assessment of the biological activity of this extract *in vivo *and on the chemical identification of the active components responsible for the anti-inflammatory activity. The knowledge of the role of flavonoïds in the immunomodulatory mechanisms in ABD is a promising area for the development of new natural's agents for the treatment of the disease and other immune-mediated diseases.

## Abbreviations

ABD: Adamantiades-Behçet's disease; Th: T helper cell; IL: interleukin: IFN-γ: interferon- γ; NO: Nitric oxide; PBMC: peripheral blood mononuclear cells; NO3*-*: nitrate; NO2*-*: nitrite; NOS2: nitric oxide synthase-2.

## Competing interests

The authors declare that they have no competing interests.

## Authors' contributions

MD carried out the experimental work, collected and interpreted the data. BH and ML carried out most of the in vivo experiments. TM and OF recruited the ABD patient's and volunteers and organized the study. BT carried out the experimental work. PY participated in the design and wrote the manuscript. TC contributed to planning of the design and execution of the project and wrote the ethic's committee application and drafting the manuscript. All authors read and approved the final manuscript.
